# Suggestions on the ideal method of conducting community screenings for older adults

**DOI:** 10.1186/s12877-023-04119-2

**Published:** 2023-06-29

**Authors:** Minoru Kouzuki, Nobuto Tanaka, Madoka Miyamoto, Katsuya Urakami

**Affiliations:** 1 Department of Biological Regulation, School of Health Science, Faculty of Medicine, Tottori University, 86 Nishi-Cho, Yonago, 683-8503 Japan; 2 Department of Dementia Prevention, School of Health Science, Faculty of Medicine, Tottori University, 86 Nishi-Cho, Yonago, 683-8503 Japan

**Keywords:** Dementia, Frailty, Screening, Cognitive function, Physical function, Olfactory function, Nutritional status

## Abstract

**Background/Objectives:**

Since dementia and frailty lead to a reduced quality of life and risk of needing long-term care in the older adults, we hypothesized that evaluations related to dementia and frailty would be useful and of high interest in screening for the older adults. Therefore, we conducted a community screening incorporating multiple simple evaluations related to dementia and frailty. In addition to various functional evaluations, we investigated interest in tests, thoughts on the disease, and the relationships between subjective (i.e., how one feels about oneself) and objective evaluations (i.e., the results of tests and rating scales). The purpose of this study was to examine the thoughts regarding tests and diseases and the functions that make it difficult to accurately perceive changes by oneself, and to obtain suggestions on the ideal method of community screening for the older adults.

**Subjects/Methods:**

The participants were 86 people aged 65 and over living in Kotoura Town who participated in the community screening, for which we obtained background information and body measurements. We also assessed physical, cognitive and olfactory function, evaluated nutritional status, and we administered a questionnaire (interest in tests, thoughts on dementia and frailty, and a subjective functional evaluation).

**Results:**

Regarding interest in tests, the participants answers were highest for physical, cognitive and olfactory function, in that order (68.6%, 60.5%, and 50.0%, respectively). In the survey on thoughts on dementia and frailty, 47.6% of participants felt that people with dementia were viewed with prejudice, and 47.7% did not know about frailty. Regarding the relationship between subjective and objective evaluations, only the assessment of cognitive function did not show a correlation between both evaluations.

**Conclusions:**

From the viewpoint of the participants’ degree of interest in and the need for accurate evaluations through objective examination, the findings suggest that the assessment of physical and cognitive function may be beneficial as a screening tool for older adults. Objective evaluation is essential, particularly for assessing cognitive function. However, approximately half the participants believed people with dementia were viewed with prejudice and did not know about frailty, which may lead to barriers to testing and low interest. The importance of increasing the participation rate in community screening through disease-related educational activities was suggested.

**Supplementary Information:**

The online version contains supplementary material available at 10.1186/s12877-023-04119-2.

## Introduction

The number of older people with dementia is estimated to increase [1] and has become a major public health problem. However, some people with mild cognitive impairment (MCI), a pre-dementia stage, revert to normal cognition [2]. In addition, a 2-year lifestyle-related multidomain intervention in a large randomized controlled trial improved cognitive function in older adults who were from the general population and were at an elevated risk of developing dementia [3]. It may be possible to prevent dementia by detecting it at the pre-disease stage and implementing interventions. Appropriate testing is necessary for early detection, and for this reason, it is important to establish a system that enables people to undergo check-ups in their local area and examinations at nearby medical institutions [4, 5]. However, in a study that conducted a questionnaire survey of the older adults, more than half of the respondents were unwilling or undecided about undergoing regular dementia screening [6]. A previous study reported that awareness of the seriousness and knowledge of a preventive lifestyle had a significant influence on the intention to undergo screening [7]. Thus, individual awareness and interest are vital factors. In addition, the stigma associated with dementia is a concern [8], and there may be resistance to participation in screening that tests cognitive function only. Various risk factors for cognitive decline and symptoms to precede cognitive decline have been suggested [9–11]. By adding evaluations other than cognitive function, it is expected that the content will attract the attention of many people, reduce resistance to participation, and lead to risk management of cognitive function decline. In recent years, in addition to dementia, frailty has been drawing attention in Japan, leading to a reduction in the quality of life and the risk of needing long-term care among the older adults. As frailty can increase the risk of dementia incidence, disability incidence, and mortality [12–17], we hypothesized that evaluations related to dementia and frailty would be useful and of high interest in screening for the older adults.

Therefore, we conducted a community screening incorporating multiple simple evaluations related to dementia and frailty, and in addition to various functional evaluations, we investigated interest in tests as well as prejudice against and awareness of prevention against dementia and frailty. Additionally, we explored the relationship between subjective and objective functional decline for each test item. By examining the presence or absence of divergence between subjective and objective evaluations, we scrutinized the necessity of objective functional evaluation by identifying items that make it difficult to accurately perceive changes. By analyzing the results, we aimed to obtain suggestions for the ideal method of community screening for the older adults, which can be carried out even in local areas where there are no specialists.

## Methods

### Participants

This study included 86 people who participated in a community screening conducted in Kotoura Town (Tottori Prefecture, Japan) from July to November of 2021. Inclusion criteria were those who lived in Kotoura Town and were over 65 years old. Exclusion criteria were those restricted from strenuous activities by their physicians owing to pre-existing medical conditions. Information about the community screening was provided on posters put up in Kotoura Town or by distributing them to residents with the cooperation of Kotoura Town Hall staff.

We conducted the present study with the approval of the Ethics Committee of the Tottori University Faculty of Medicine (No. 20A227). Prior to conducting the research, the participants were informed about the study’s aims and their consent was obtained in writing.

### Community screening measurement items

#### Participant demographic characteristics

Age, sex, years of education, number of drugs taken, and medical history (hypertension, dyslipidemia, diabetes mellitus, olfactory disorder with obvious cause, and locomotive organ disorder) were investigated through interviews.

#### Body measurements

Height was measured using a tape measure attached to the wall and weight, body fat percentage, basal metabolic rate, body water percentage, and muscle mass were measured using a weight and body composition monitor with dual frequency bioelectrical impedance analysis (RD-800; TANITA Corporation, Tokyo, Japan). From the results, we computed body mass index ((BMI) = weight (kg) / height^2^ (m^2^)) and appendicular skeletal muscle mass index ((SMI) = arm and leg skeletal muscle mass (kg) / height^2^ (m^2^)).

#### Assessment of cognitive function

To assess cognitive function, we used an existing computerized test battery for Alzheimer’s disease screening (developed by Nihon Kohden Corporation, Tokyo, Japan, with the product name “*monowasure soudan proguramu* (MSP)” (which means forgetfulness consultation program)) [18]. In the test, the participants performed on their own, following the instructions given by the computer (MSP-1100; Nihon Kohden Corporation, Tokyo, Japan). It comprises four evaluation items: a three-word memory test, a temporal orientation test, a 3D visual-spatial perception test, and a delayed recall test. The maximum score was 15 points for answering all questions correctly and the minimum score was 0 points for answering all questions incorrectly. The sensitivity and specificity for distinguishing between healthy controls and Alzheimer’s disease were 96% and 86%, respectively, and the cutoff value was set to 12/13 points [18].

#### Assessment of olfactory function

Olfactory function may be impaired prior to cognitive decline [10], hence it may be useful for the early detection of dementia. Olfactory function was assessed using the Open Essence (OE) test (FUJIFILM Wako Pure Chemical Corporation, Osaka, Japan) [19–21]. The OE test included 12 odor items. Each odorant was contained in 12 cards and folded in two. In the center of the left half of the open card, microencapsulated test odorants were applied instead of glue. On the other side, six alternatives (four alternatives for odor name, as well as “detectable but not recognizable” and “no smell detected”) were printed. Participants opened the odor cards one by one in order, sniffed them, and identified the odorant among 6 choices. A score of 1 point per odor and a score of 12 points indicated that the participant had answered all questions correctly, and a score of 0 indicated that the participant’s answers were all incorrect. It has been shown that setting a cut-off value of the OE test of 7 points or less as a screening criterion for olfactory disorders is useful for differentiation [21].

#### Assessment of physical function

Physical function tests included evaluation of grip strength, balance, gait speed, the chair sit-stand test, and the Timed Up and Go test (TUG) [22]. We also calculated the short physical performance battery (SPPB) score [23] based on the results of the balance, gait speed, and chair sit-stand tests. On the SPPB, the participant can gain a maximum of 12 points (0 to 4 points for each of the three items), and a higher score denotes better physical performance. An SPPB score of ≤ 9 points is considered useful for predicting mortality [24], and in this study, individuals with a score of ≤ 9 points were judged to have low SPPB scores.

Grip strength was evaluated using a hand dynamometer (T.K.K.5401; Takei Scientific Instruments Co. Ltd., Niigata, Japan). Measurements were taken once on each side in a standing position; we used the results from the strongest hand in the present analysis.

We assessed balance strength for the time required to keep standing in the side-by-side, semi-tandem, and full tandem positions. The maximum measurement time was 10 s in each standing position, and the full tandem position was performed only when the participant could hold both the side-by-side and semi-tandem positions for 10 s.

Gait speed was assessed by measuring the walking time at 4 m and calculating the gait speed for 1 s. To reproduce a situation close to normal walking, the participant was asked to walk 6 m with an additional 1 m each before and after the 4 m measurement point.

In the chair sit-stand test, participants were asked to sit down on a chair, fold their arms in front of their chest, and repeat five consecutive sit-to-stand motions from a sitting position as quickly as possible. The time required to complete the fifth standing movement from the start of the movement was measured.

The TUG test began from sitting with the back against the back of the chair, standing up from the chair, walking to a landmark 3 m ahead, turning around, and measuring the time it took to sit back in the chair. The results of the maximum walking speed were used in the analysis.

#### Assessment of frailty

We assessed physical frailty using the Japanese version of the Cardiovascular Health Study criteria [25]. These criteria comprise five domains: shrinking, low activity, exhaustion, weakness, and slowness. (i) Shrinking: Have you lost 2 kg or more in the past 6 months? (“Yes” is one point); (ii) low activity: (a) Do you engage in moderate levels of physical exercise or sports aimed at health? (b) Do you engage in low levels of physical exercise aimed at improving health? (“No” to both questions is one point); (iii) exhaustion: In the past 2 weeks, have you felt tired without a reason? (“Yes” is one point); (iv) weakness: grip strength < 28 kg in men or < 18 kg in women (one point if there is a decrease); and (v) slowness: gait speed < 1.0 m/s (one point if there is a decrease). Participants with none of these components were considered robust, those with one or two components were considered to be pre-frailty, and those with three or more components were considered frailty [25].

#### Assessment of sarcopenia and dynapenia

We diagnosed sarcopenia and dynapenia based on the Asian Working Group for Sarcopenia 2019 criteria and Yamada et al. [26, 27]. Muscle functional decline was defined as a grip strength of < 28 kg in men or < 18 kg in women and/or gait speed of < 1.0 m/s, and reduced muscle mass was defined as an SMI of < 7.0 kg/m^2^ in men or < 5.7 kg/m^2^ in women.

Sarcopenia was defined as low muscle mass and low muscle function; pre-sarcopenia was defined as low muscle mass without low muscle function; dynapenia was defined as low muscle function without low muscle mass; and normal was defined as anything other than the above.

#### Nutritional related assessment

Nutritional assessment included evaluation of dietary variety using the dietary variety score (DVS) developed by Kumagai et al. [28] and the Mini Nutritional Assessment-Short Form (MNA®-SF) [29, 30].

The DVS is an assessment that investigates the weekly frequency of intake of 10 food-based components, including fish and shellfish, meat, eggs, milk, soybean products, green and yellow vegetables, seaweed, potatoes, fruits, fat, and oil. The total DVS ranges from 0 to 10 points, with the intake of each food group assigned 1 point for a response of “eat almost every day” and 0 for “eat once every two days,” “eat once or twice a week,” or “eat hardly ever.” A previous study evaluated DVS among 608 community-dwelling residents and found that the 10^th^ percentile of DVS scores for all subjects was ≤ 3 points [28]. Hence, in this study, a score of 3 or less was considered to indicate low diversity in food intake.

The MNA®-SF consists of six items: reduction in food intake over the past three months, weight loss during the past three months, mobility, psychological stress or acute disease in the past three months, neuropsychological problems, and BMI. The score ranges from 0 to 14 and is interpreted as follows: 12–14 indicates normal nutritional status, 8–11 denotes a risk of malnutrition, and 0–7 means malnourished [30].

#### Questionnaire

We conducted a questionnaire on interest in the test, the subjective state assessment, and dementia- and frailty-related items.

For the tests of interest, participants were asked, "please check the evaluation that you are interested in the items of this community screening," and selected from five items: body measurements (e.g., height and weight measurements), the cognitive function test, the olfactory function test, and the physical function test (e.g., grip strength, lower limb muscle strength, balance strength, body composition measurement), and evaluation of nutritional status. The questionnaire allowed for multiple responses.

As a subjective condition questionnaire, we investigated the perception of functional decline in six areas: decline in cognitive function, decline in olfactory function, decline of upper extremity muscles, decline of leg muscles, decline in physical function, and decline in nutritional status. Participants were asked to respond to a question about each decline in function using four options: “strongly disagree”, “disagree,” “agree,” and “strongly agree.” We instructed them to answer “disagree” if they felt that it was appropriate for their age.

We investigated the degree of social prejudice and interest in prevention by using questions on dementia and frailty. Regarding dementia-related questions, we asked whether people with the disease were viewed with prejudice, along with their thoughts about prevention (motivation for preventive activities at home, willingness to participate in preventive care projects held by municipalities, the presence/absence of current preventive activities, and thoughts on the effectiveness of preventive efforts). With regard to frailty, in addition to the above questions, we asked about understanding the definition. If they answered “I don't know” to the question about the definition of frailty, they were asked not to answer questions about prejudice against and the prevention of frailty.

### Statistical analysis

We performed statistical analysis using the software SPSS version 27 (IBM Corporation, Tokyo, Japan). We used the Shapiro–Wilk test to assess the normality of the data, and Levene’s test to assess the equality of variance. We employed univariate and multivariate binary logistic regression analyses to establish associations between whether participants felt that people with dementia and frailty were viewed with prejudice (the dependent variable) and background factors (e.g., age, sex, years of education, and number of drugs as independent variables). We classified those who answered “agree” or “somewhat agree” to questions about whether people with dementia and frailty are perceived with prejudice as the group that felt social prejudice. In addition, we used one of the four items (age, gender, years of education, and number of drugs) as the target factor to investigate the effect, and we adjusted the remaining three items as covariates. However, 14 people answered that they agreed with the question “Do you think people with frailty tend to be viewed with prejudice?” As the validity of the logistic model becomes problematic when the ratio of the number of events per variable analyzed is small [31], we did not perform a covariate-adjusted statistical analysis of the association between background factors and prejudice against frailty. In the logistic regression analysis, we calculated the unadjusted and adjusted odds ratios (OR) and 95% confidence intervals (CIs). Regarding the link between the subjective and objective functional evaluations, we divided responses regarding subjective function into “strongly agree:4 points,” “agree:3 points,” “disagree:2 points,” and “strongly disagree:1 point.” We used tests or existing questionnaires with established usefulness, such as the DVS and MNA®-SF, for objective evaluation. In other words, in this study, we defined subjective evaluation as how participants feel about themselves and objective evaluation as the results of tests and rating scales. We determined the association between the subjective and objective functional evaluations by calculating Spearman’s rank correlation coefficient. In addition, we made comparisons of various test outcomes between groups with high and low scores on cognitive function tests using Student’s t-test, Welch’s t-test, or the Mann–Whitney U test.

All statistical significance tests were two-sided, and an alpha level of 0.05 was statistically significant.

## Results

### Characteristics of the participants

Table 1 presents the characteristics of the participants. The mean age was 77.3 (standard deviation: 6.7) years, 87.2% were female; and the mean years of education was 11.3 (standard deviation: 1.8) years. The prevalence of cognitive decline was 12.8%. The rate of pre-frailty and frailty were 35.5% and 3.9%, respectively. Regarding other results, notable characteristic was that 70.7% of participants were judged to have olfactory disorder, which is a high percentage. Table 2 shows the results of the subjective functional assessments. A fairly large number of participants—more than 60%—responded that they “strongly agree” or “agree” with experiencing a decline in cognitive function, a decline of the upper extremity muscles, a decline of leg muscle, and a decline in physical function (61.6%, 75.6%, 62.8%, and 72.1%, respectively).

**Table 1:** Characteristics of the participants

	All participants (*n* = 86)
Age (years)	77.3 ± 6.7
Gender	
Male	11 (12.8)
Female	75 (87.2)
Education (years)	11.3 ± 1.8
Number of drugs	2.2 ± 2.0
Medical history	
Hypertension	41 (47.7)
Dyslipidemia	32 (37.2)
Diabetes mellitus	10 (11.6)
Olfactory disorder	3 (3.5)
Locomotive organ disorder	21 (24.4)
BMI (kg/m^2^)	22.5 ± 3.1
MSP (points)	13.9 ± 1.6
Non-cognitive decline (MSP≧13)	75 (87.2)
Cognitive decline (MSP≦12)	11 (12.8)
OE (points)^a^	5.8 ± 2.7
Non-olfactory disorder (OE≧8)	24 (29.3)
Olfactory disorder (OE≦7)	58 (70.7)
Grip strength (kg)^b^	
Male	34.6 ± 6.2
Female	21.4 ± 4.1
Balance test (sec)^c^	
Side-by-side stand	10.0 ± 0
Semi-tandem stand	10.0 ± 0.4
Full tandem stand	9.5 ± 1.5
Gait speed (m/sec)^d^	1.2 ± 0.3
Chair sit-stand test (sec)^e^	9.8 ± 4.7
SPPB (points)^e^	11.4 ± 1.1
High score (SPPB≧10)	68 (93.2)
Low score (SPPB≦9)	5 (6.8)
TUG (sec)^e^	7.1 ± 2.0
SMI (kg/m^2^)^f^	
Male	7.5 ± 0.8
Female	6.5 ± 0.8
Body fat percentage (%)	29.2 ± 7.8
Basal metabolic rate (kcal)	1022.3 ± 191.7
Body water percentage (%)	49.4 ± 5.4
Frailty status^g^	
Robust	46 (60.5)
Pre-frailty	27 (35.5)
Frailty	3 (3.9)
Sarcopenia and dynapenia staging^h^	
Normal	52 (70.3)
Dynapenia	14 (18.9)
Presarcopenia	1 (1.4)
Sarcopenia	7 (9.5)
MNA®-SF (points)	12.4 ± 1.4
Normal nutritional status	68 (79.1)
At risk of malnutrition	18 (20.9)
Malnourished	0 (0)
DVS (points)	5.0 ± 2.5
Not low score (DVS≧4)	60 (69.8)
Low score (DVS≦3)	26 (30.2)

Data presented as mean ± standard deviation or number (%)

^a^Sample size is 82

^b^Sample size is 76 (9 men / 67 women)

^c^Sample size is 75

^d^Sample size is 77

^e^Sample size is 73

^f^Sample size is 75 (9 men / 66 women)

^g^Sample size is 76

^h^Sample size is 74

*BMI* Body mass index, *MSP* a computerized test battery for Alzheimer’s disease screening (produced by Nihon Kohden Corporation, called *monowasure soudan proguramu* (forgetfulness consultation program)), *OE* Open essence, *SPPB* Short physical performance battery, *TUG* Timed Up and Go Test, *SMI* Skeletal muscle mass index, *MNA®-SF* Mini Nutritional Assessment-Short Form, *DVS* Dietary variety score

**Table 2:** Results of subjective functional assessment

	Number (%)
Decline in cognitive function	
Strongly disagree	5 (5.8)
Disagree	28 (32.6)
Agree	45 (52.3)
Strongly agree	8 (9.3)
Decline in olfactory function	
Strongly disagree	23 (26.7)
Disagree	36 (41.9)
Agree	19 (22.1)
Strongly agree	8 (9.3)
Decline of upper extremity muscles	
Strongly disagree	3 (3.5)
Disagree	18 (20.9)
Agree	49 (57.0)
Strongly agree	16 (18.6)
Decline of leg muscle	
Strongly disagree	6 (7.0)
Disagree	26 (30.2)
Agree	37 (43.0)
Strongly agree	17 (19.8)
Decline in physical function	
Strongly disagree	3 (3.5)
Disagree	21 (24.4)
Agree	49 (57.0)
Strongly agree	13 (15.1)
Decline in nutritional status	
Strongly disagree	20 (23.3)
Disagree	46 (53.5)
Agree	18 (20.9)
Strongly agree	2 (2.3)

### Tests of interest

**Fig. 1:**
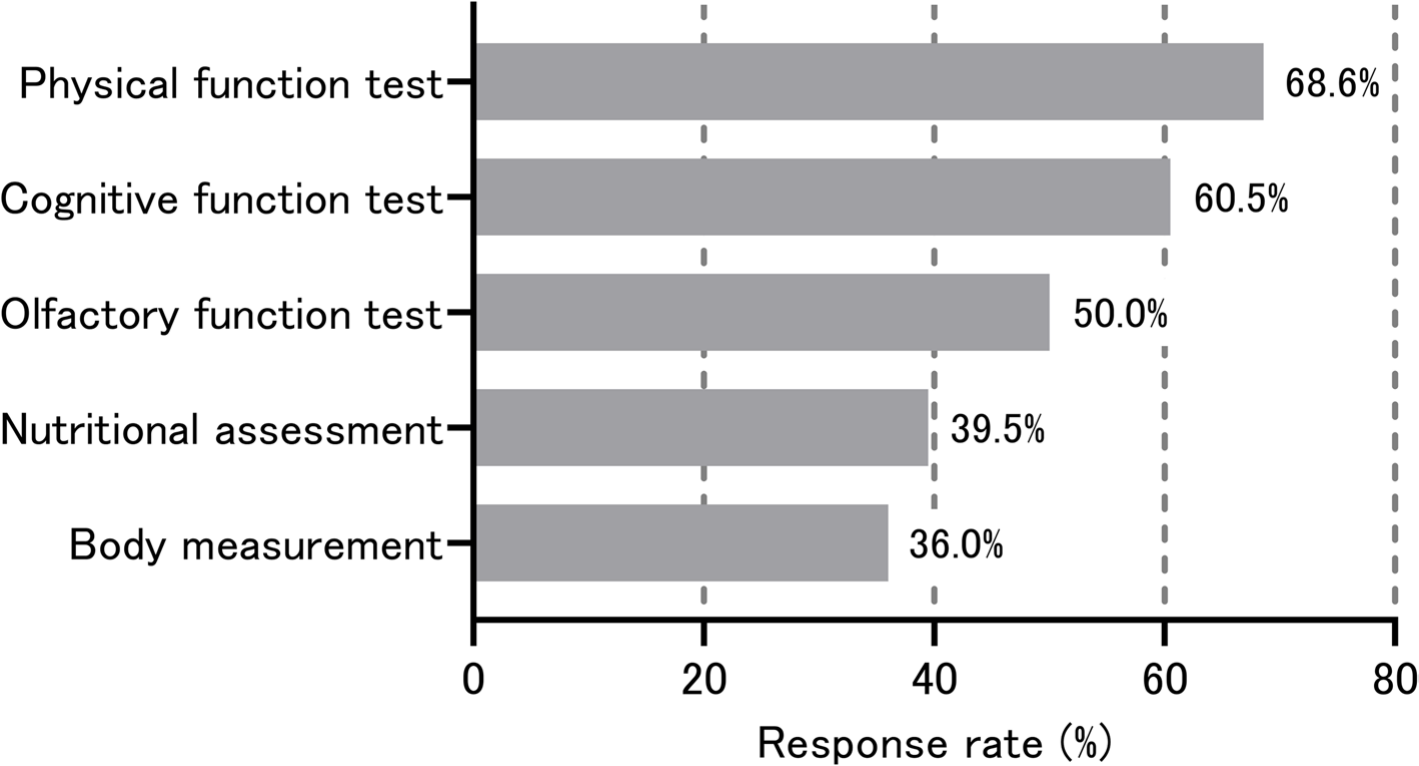
Questionnaire results about tests of interest

Figure 1 presents the results of the questionnaire on tests of interest. Items of interest were, from the highest percentage, the physical function test (68.6%), the cognitive function test (60.5%), the olfactory function test (50.0%), nutritional status (39.5%), and body measurements (36.0%). In addition, 82.6% of the participants answered that they were interested in either the physical or cognitive function test, with a rate of 90.7% when the olfactory function test was added to the above two items, 84.9% when nutritional status was added to the above two items, and 88.4% when the aspect of body measurements was added to the above two items.

### Questionnaire results regarding prejudice against, and prevention of, dementia and frailty

Table 3 depicts the outcomes regarding prejudice against, and prevention of, dementia and frailty. The results of prejudice against, and prevention of, frailty were responses from 45 people, excluding 41 (47.7%) who did not know about frailty. Participants answered “agree” or “somewhat agree” to questions about whether people with dementia and frailty are viewed with prejudice (47.6% [41 out of 86] for dementia and 31.1% [14 out of 45] for frailty). We performed logistic regression analyses to examine the factors related to the degree of social prejudice against dementia or frailty (Table 4). Age (OR = 0.90, 95% CI = 0.82–0.99, *p* = 0.030) and years of education (OR = 1.46, 95% CI = 1.03–2.06, *p* = 0.034) were significantly associated with the feeling that people with dementia are viewed with prejudice in the adjusted model. In other words, younger people or those with more years of education were more likely to feel that people with dementia were viewed with prejudice. As for the prevention of dementia and frailty, many participants wanted to work on a simple preventive method that could be performed at home: with 79.1% for dementia prevention and 82.2% for frailty prevention. However, very few participants thought that efforts to prevent dementia and frailty would be effective, with 11.6% for dementia prevention and 2.2% for frailty prevention.

**Table 3:** Questionnaire results regarding prejudice against, and prevention of, dementia and frailty

	Number (%)
Do you think people with dementia tend to be viewed with prejudice?	
Agree	10 (11.6)
Somewhat agree	31 (36.0)
Somewhat disagree	17 (19.8)
Disagree	28 (32.6)
What are your thoughts on the prevention of dementia? (multiple answers allowed)	
If there is a simple preventive method that can be done at home, I would like to work on it	68 (79.1)
I would like to participate in preventive care projects held by municipalities	42 (48.8)
I am already doing some kind of preventive activity	33 (38.4)
I do not think we can expect much of an effect from dementia prevention efforts	10 (11.6)
Do you know what the term *fureiru* (frailty in English) refers to?	
Healthy condition	2 (2.3)
State of weakness	33 (38.4)
State of need for long-term care	10 (11.6)
I do not know	41 (47.7)
Do you think people with frailty tend to be viewed with prejudice?^a^	
Agree	1 (2.2)
Somewhat agree	13 (28.9)
Somewhat disagree	17 (37.8)
Disagree	14 (31.1)
What are your thoughts on the prevention of frailty? (multiple answers allowed)^a^	
If there is a simple preventive method that can be done at home, I would like to work on it	37 (82.2)
I would like to participate in preventive care projects held by municipalities	24 (53.3)
I am already doing some kind of preventive activity	22 (48.9)
I do not think we can expect much of an effect from frailty prevention efforts	1 (2.2)

^a^The results of responses from 45 people, excluding those who answered “I don’t know” to the question about the definition of frailty are shown

**Table 4:** Analysis of factors influencing whether participants feel social prejudice against people with dementia or frailty

	Unadjusted		Adjusted^b^	
	OR (95% CI)	*P* value	OR (95% CI)	*P* value
Association with prejudice against dementia				
Age	0.86 (0.79—0.94)	0.001	0.90 (0.82—0.99)	0.030
Gender (Female)	0.73 (0.21—2.60)	0.626	0.96 (0.23—4.07)	0.952
Education	1.74 (1.27—2.38)	0.001	1.46 (1.03—2.06)	0.034
Number of drugs	0.86 (0.69—1.08)	0.196	1.01 (0.78—1.30)	0.959
Association with prejudice against frailty^a^				
Age	1.00 (0.89—1.13)	0.976	-^c^	
Gender (Female)	0.54 (0.10—2.84)	0.469	-^c^	
Education	1.12 (0.78—1.60)	0.549	-^c^	
Number of drugs	1.34 (0.92—1.97)	0.133	-^c^	

^a^The results of responses from 45 people, excluding those who answered “I don’t know” to the question about the definition of frailty are shown

^b^Analysis was performed using one of the four items (age, gender, education, and number of drugs) as the independent variable and the remaining three as adjustment covariates

^c^Fourteen people agreed with the question, “Do you think people with frailty tend to be viewed with prejudice?” Because the validity of the logistic model becomes problematic when the ratio of the number of events per variable analyzed is small, statistical analysis was not performed

OR, odds ratio; CI, confidence interval

### The correlation between the subjective and objective evaluations

Table 5 portrays the outcomes of the correlation analysis between the subjective and objective evaluation items that are considered to be related. Except for cognitive function, the results of one or another objective assessment revealed a significant correlation with the subjective assessment. Concerning cognitive function, the results indicated that even participants with high scores on the cognitive function test often complained of declining cognitive function (Additional File 1: Table S1). In addition, participants with lower scores on cognitive function tests performed significantly worse on the balance test (semi-tandem stand), gait speed, SPPB, and TUG tests than those with higher scores (Additional file 1: Table S2).

**Table 5:** Correlation between subjective and objective evaluations

			Subjective evaluation based on the questionnaire					
Test item	Sample size	If fluctuations match	Cognitive function	Olfactory function	Upper extremity muscles	Leg muscles	Physical function	Nutritional status
MSP	86	Negative correlation	-0.053					
OE	82	Negative correlation		-0.227 *				
Grip strength								
Male	9	Negative correlation			-0.798 *		-0.329	
Female	67	Negative correlation			-0.130		-0.068	
Balance test								
Side-by-side stand	75	Negative correlation				- ^a^	- ^a^	
Semi-tandem stand	75	Negative correlation				-0.058	-0.042	
Full tandem stand	75	Negative correlation				-0.205	0.018	
Gait speed	77	Negative correlation				-0.336 *	-0.241 *	
Chair sit-stand test	73	Positive correlation				0.233 *	0.193	
SPPB	73	Negative correlation				-0.291 *	-0.064	
TUG	73	Positive correlation				0.454 *	0.325 *	
SMI								
Male	9	Negative correlation			0.222	0.062	0.299	
Female	66	Negative correlation			-0.033	0.094	0.036	
BMI	86	Negative correlation						-0.226 *
MNA®-SF	86	Negative correlation						-0.236 *
DVS	86	Negative correlation						-0.143

The data are presented as correlation coefficients. All correlation analyses were conducted using Spearman’s correlation coefficients

Only correlation results with objective evaluations considered relevant to each subjective evaluation are shown

^a^Statistical analysis was not possible because the results for side-by-side standing times were the same for all participants

^*^p < 0.05

*MSP* a computerized test battery for Alzheimer’s disease screening (produced by Nihon Kohden Corporation, called *monowasure soudan proguramu* (forgetfulness consultation program)), *OE* Open essence, *SPPB* Short physical performance battery, *TUG* Timed Up and Go Test, *SMI* Skeletal muscle mass index, *BMI* Body mass index, *MNA®-SF* Mini Nutritional Assessment-Short Form, *DVS* Dietary variety score

## Discussion

Among community residents aged 65 and over who participated in the community screening, the percentage of respondents who expressed interest in physical or cognitive function tests were more than 60%, and more than 80% were interested in either physical or cognitive function tests. These results indicate a high level of interest in cognitive and/or physical function tests. Moreover, 12.8% of the participants had cognitive decline, 3.9% were frailty, and 35.5% were pre-frailty. Several cross-sectional studies in Japan conducted on community-dwelling with individuals over 65 years old [32–37] was reported that the rate of cognitive impairment was 2.0–10.8% in the urban areas and 8.4–21.8% in the rural areas, and the rates of frailty and pre-frailty were 8.7–12.8% and 35.4–47.7%, respectively. There are some differences in results due to regional differences, differences in sociodemographic characteristics, and differences in evaluation methods. However, potentially there are people with physical and cognitive decline in the community. The importance of objective evaluations can be evident in the fact that more than 60% of participants felt subjectively impaired in their physical and cognitive functions. Furthermore, by conducting other tests in addition to the physical and cognitive function tests, the rate of interest in the test will increase, which is expected to improve the participant rate; it is also meaningful in terms of knowing the risk of various diseases. In particular, adding an olfactory test could attract more than 90% of participants. Since olfactory function may be impaired prior to cognitive decline [10], it is highly valuable to introduce it into community screenings. However, in this study, the percentage below the cut-off value in the olfactory test was very high at 70.7% in this study. Olfactory function declines with age [38, 39], and a previous study that conducted OE in community-dwelling older adults with a mean age of 73 years also demonstrated a high proportion (67.4%) with impaired olfactory function [37]. Thus, it is necessary to understand the high detection rate of decreased olfactory function when conducting olfactory tests in community screenings for the older adults. In addition, since olfactory impairment may precede a decline in cognitive function [10], we believe that it is important to conduct a longitudinal evaluation of people with reduced olfactory function but without reduced cognitive function.

Except for cognitive function, the results of one or more objective assessments revealed a significant correlation with the subjective assessment; as such, even a simple questionnaire survey can capture the state to some extent. Therefore, we believe that this can be simplified for the evaluation of physical functions that are of great interest. However, we assessed multiple items for physical function, and if it was difficult to spend time and effort, we believe it is acceptable to conduct a questionnaire survey and additionally select simple objective evaluation items such as grip strength and walking speed, which are related to the judgment of physical frailty [25], or items that showed significant deterioration in cognitively impaired participants (Additional file 1–Table S2). However, regarding cognitive function, the results indicated that even participants with high scores on the cognitive function test often complained of declining cognitive function. Nogi et al. also reported that older adults with or without cognitive dysfunction were excessively aware of memory loss [40]. Regarding cognitive function, the objective evaluation is necessary, regardless of the presence or absence of subjective symptoms.

From a survey of thoughts about dementia, we found that approximately half the participants felt that people with dementia were viewed with prejudice. There is a possibility that some participants felt that people with dementia tended to be viewed with prejudice from public impressions, even if they themselves did not have prejudice against people with dementia. Therefore, this finding should be interpreted with caution. However, the results can be interpreted as reflecting the social climate of prejudice against dementia, as they indicate that participants had prejudice-related experiences directly or indirectly. The results showed that social prejudice against people with dementia remains highly prevalent among Japanese people. In addition, younger age or more years of education was associated with the feeling that people with dementia are viewed with prejudice. This is similar to a previous study showing that respondents aged 75 years or older expressed less perceived stigma than younger respondents [41]. There may be various factors that explain this, but we think that younger people tend to have prejudices because they have few opportunities to interact with people with dementia and feel it is someone else’s problem. Moreover, people may tend to accept being diagnosed with dementia as they age. In contrast, a study conducted in 2008 on older Korean Americans indicated that feelings of shame associated with family members having Alzheimer’s disease are more likely to be reported by individuals with lower levels of education and less knowledge of Alzheimer’s disease [42]. Although the countries, years, and methods surveyed were different, the results differed. Previous investigations have suggested that a higher level of education may be associated with more knowledge of dementia [42, 43]. However, based on our results, we speculate that people with a high level of education may have acquired a certain amount of knowledge, but at the same time, they may have acquired more incorrect information, making them more likely to have prejudice. As health professionals expressed the highest levels of stigma compared to other groups (social workers, students, retired people, and the public) [41], it is possible that a high level of knowledge does not simply mean less prejudice. Furthermore, we asked whether people with dementia tend to be viewed with prejudice. Thus, it is also conceivable that more knowledgeable people may have a higher awareness of current social climate — such as the stigma associated with dementia, which is a concern [8]. However, we have not been able to find any past research results that support our view; therefore, we should be careful in interpreting this because of our unsubstantiated opinion. In the future, we think it is necessary to examine whether there are cultural differences and historical backgrounds in prejudice against dementia. In addition, there is a need to accurately identify the factors associated with prejudice by examining the presence or absence of additional factors, such as modifiable acquired variables associated with prejudice, and using reliable methods to assess the presence of prejudice. Prejudice regarding the disease can be a barrier to testing. However, appropriate interventions can enhance the public’s knowledge of dementia and reduce dementia-related stigma, especially for those with higher levels of stigma [44]. We think it is necessary to provide correct information, even to young people, and to encourage a change in awareness so that prejudice can be eliminated.

Regarding frailty, in a previous study that surveyed a community-dwelling older population in Japan in 2018, awareness of the term *frailty* was estimated at 20.1% [45]. In contrast, in this study, 38.4% of the participants correctly understood the definition. Degree of recognition seems to be increasing little by little, but 13.9% of the participants misunderstood, and 47.7% of the participants answered that they did not know. From the above, we believe that the term is not yet well-known among older adults. We assert that increasing the degree of recognition of frailty by disseminating correct information will heighten interest in testing.

In addition, many people believe that dementia and frailty can be prevented, and many people would like to work on doing so if there is a simple preventive method that can be performed at home. Farming, exercise, intellectual activity, and social participation help improve and prevent frailty [46], and certain active lifestyles may contribute to the return of cognitive function to normal levels after MCI [47]. In other words, many actions can be taken in daily life to prevent dementia and frailty. We affirm that it is important to establish a system that provides tests as well as post-test measures.

This study has several limitations. First, the participants were those who voluntarily participated in a community screening for the older adults conducted in Kotoura Town. A previous study reported that non-participation in a physical checkup may be related to poor health awareness [48]. Therefore, it is possible that there was a sampling bias in that many of the participants were highly health conscious. Second, nearly 90% of the participants were female. As such, our data might reflect the results for women in particular. Third, information on sociodemographic characteristics was scarce. Therefore, it was not possible to interpret the results by considering the characteristics of the participants such as their occupation, marital status, lifestyle, and income. Additionally, the lack of detailed participant characteristics may make comparisons with other studies difficult. The fourth limitation is the small sample size. In addition, there were missing values for many evaluation items; therefore, the number of subjects for the analysis was even smaller. We believe that an examination with a larger sample size is required to draw more accurate conclusions.

## Conclusions

Because there are a certain number of people in the community who have cognitive decline and frailty or a prodromal state (pre-frailty), it is important to conduct community screenings and evaluate them. Regarding the content of community screening for the older adults, from the viewpoint of the participants’ degree of interest and the need for accurate objective evaluation, the evaluation of physical and cognitive functions may be beneficial. Cognitive function cannot be accurately judged by oneself; therefore, an objective examination is essential. However, regarding physical function, we found a relationship between subjective symptoms and test results. If it is difficult to spend the time and effort, we believe this can be simplified. Furthermore, by conducting other tests in addition to physical and cognitive function tests, the rate of interest in testing will increase, which is expected to make community screening attractive to many people. The results of this study will be helpful as objective information for selecting test items for community screening of older individuals. However, there is a concern that approximately half of the participants felt that people with dementia were viewed with prejudice and did not know about frailty, which may lead to barriers regarding testing and low interest. The results of this study indicate that community screening for the older adults to conduct evaluations related to dementia and frailty is useful and of high interest, but also suggest the importance of increasing the participation rate in community screening through disease-related educational activities.

## Supplementary Information

**Additional file 1.**

## Data Availability

The datasets used and/or analyzed during the current study are available from the corresponding author upon reasonable request, subject to the approval of all authors and the ethics committee.

## Abbreviation

BMI: Body mass index
MSP: A computerized test battery for Alzheimer’s disease screening (produced by Nihon Kohden Corporation, and product name is monowasure soudan proguramu (MSP) (it means forgetfulness consultation program))
OE: Open essence
SPPB: Short Physical Performance Battery
TUG: Timed Up and Go test
SMI: Skeletal muscle mass index
MNA®-SF: Mini Nutritional Assessment-Short Form
DVS: Dietary variety score
OR: Odds ratio
CI: Confidence interval
MCI: Mild cognitive impairment
