# Supplementary material for: Suggestions on the ideal method of conducting community screenings for older adults
Source: BMC Geriatr. 2023 Jun 29;23:397. doi: 10.1186/s12877-023-04119-2 (PMC10308771; doi:10.1186/s12877-023-04119-2)
Supplement: Supplementary file 1 — Additional file 1. [file 12877_2023_4119_MOESM1_ESM.docx]

**Additional file 1**

Table S1. Correlation between subjective and objective evaluations of cognitive function

|  | Do you feel cognitive decline? (n, %) | | | |
| --- | --- | --- | --- | --- |
|  | Strongly  agree | Agree | Disagree | Strongly  disagree |
| MSP cutoff value is 13/12 points |  |  |  |  |
| High cognitive function test scores (n=75) | 7 (9.3) | 40 (53.3) | 23 (30.7) | 5 (6.7) |
| Low cognitive function test scores (n=11) | 1 (9.1) | 5 (45.5) | 5 (45.5) | 0 (0) |

MSP is a computerized test battery for Alzheimer’s disease screening (produced by Nihon Kohden Corporation, called *monowasure soudan proguramu* (forgetfulness consultation program)).

Table S2. Comparison between the cognitive function test and other tests

|  | MSP ≧ 13 points | |  | MSP ≦ 12 points | |  |  |
| --- | --- | --- | --- | --- | --- | --- | --- |
| Test item | Sample size | Mean ± SD |  | Sample size | Mean ± SD |  | P value |
| OE | 72 | 6.0 ± 2.7 |  | 10 | 4.9 ± 2.8 |  | 0.222 |
| Grip strength |  |  |  |  |  |  |  |
| Male | 9 | 34.6 ± 6.2 |  | 0 | - |  | - ^a^ |
| Female | 57 | 21.8 ± 4.1 |  | 10 | 19.1 ± 3.3 |  | 0.052 |
| Balance test |  |  |  |  |  |  |  |
| Side-by-side stand | 66 | 10.0 ± 0 |  | 9 | 10.0 ± 0 |  | - ^b^ |
| Semi-tandem stand | 66 | 10.0 ± 0 |  | 9 | 9.6 ± 1.1 |  | 0.007 |
| Full tandem stand | 66 | 9.6 ± 1.5 |  | 9 | 9.1 ± 1.8 |  | 0.235 |
| Gait speed | 66 | 1.3 ± 0.3 |  | 11 | 1.0 ± 0.3 |  | 0.009 |
| Chair sit-stand test | 64 | 9.6 ± 4.9 |  | 9 | 10.9 ± 3.2 |  | 0.092 |
| SPPB | 64 | 11.6 ± 0.9 |  | 9 | 10.4 ± 1.4 |  | 0.004 |
| TUG | 64 | 6.8 ± 1.7 |  | 9 | 8.9 ± 2.8 |  | 0.019 |
| SMI |  |  |  |  |  |  |  |
| Male | 9 | 7.5 ± 0.8 |  | 0 | - |  | - ^a^ |
| Female | 56 | 6.5 ± 0.7 |  | 10 | 6.9 ± 1.3 |  | 0.335 |
| BMI | 75 | 22.4 ± 2.9 |  | 11 | 23.5 ± 4.5 |  | 0.273 |
| MNA®-SF | 75 | 12.4 ± 1.3 |  | 11 | 12.5 ± 1.6 |  | 0.984 |
| DVS | 75 | 5.1 ± 2.5 |  | 11 | 3.9 ± 2.0 |  | 0.102 |

Data are presented as the mean ± standard deviation. SMI in females was compared using Welch’s t-test. Grip strength in females, gait speed, and BMI were compared using Student’s t-test. Items other than those above were compared using the Mann–Whitney U test.

^a^ Statistical analysis was not possible because none of the participants had an MSP score of 12 or less.

^b^ Statistical analysis was not possible because the results for side-by-side stand time were the same for all participants.

MSP, a computerized test battery for Alzheimer’s disease screening (produced by Nihon Kohden Corporation, called *monowasure soudan proguramu* (forgetfulness consultation program)); OE, open essence; SPPB, short physical performance battery; TUG, Timed Up and Go Test; SMI, skeletal muscle mass index; BMI, body mass index; MNA®-SF, Mini Nutritional Assessment-Short Form; DVS, dietary variety score; SD, standard deviation.
